# Immunogenicity and Safety of the AS01_E_-adjuvanted Respiratory Syncytial Virus (RSV) Prefusion F Protein Vaccine in Adults Aged 18–49 Years at Increased Risk of RSV Disease Compared with Adults Aged ≥60 Years

**DOI:** 10.1093/cid/ciag058

**Published:** 2026-02-27

**Authors:** Essack Mitha, Murdo Ferguson, Agatha Cathrine Wilhase, Ferdinandus De Looze, Marie-Louise Vachon, Helen Stacey, Roy Rasalam, Minoru Nozaki, William B Smith, Tino F Schwarz, Hiwot Amare Hailemariam, Quentin Deraedt, Catherine Gérard, Carline Vanden Abeele, Silvia Damaso, Dominique Descamps, Judith Hill, Dileep Dasyam, Jonathan Van Gucht, Veronica Hulstrøm

**Affiliations:** Newtown Clinical Research Centre, Johannesburg, South Africa; Colchester Research Group, Truro, Canada; REIMED Pty., Boksburg, South Africa; Momentum Clinical Research Wellers Hill, Brisbane, Australia; Centre de Recherche du Centre Hospitalier Universitaire de Québec-Université Laval, Québec, Canada; Diablo Clinical Research, Walnut Creek, California, USA; Momentum Clinical Research Sunshine, Melbourne, Australia; University of Melbourne, Melbourne, Australia; Shirayurikai Swing Nozaki Clinic, Tokyo, Japan; AMR Clinical, Knoxville, Tennessee, USA; Institute of Laboratory Medicine and Vaccination Centre, Klinikum Würzburg Mitte, Juliusspital, Würzburg, Germany; GSK, Wavre, Belgium; GSK, Wavre, Belgium; GSK, Rixensart, Belgium; GSK, Wavre, Belgium; GSK, Wavre, Belgium; GSK, Wavre, Belgium; GSK, Wavre, Belgium; GSK, Wavre, Belgium; GSK, Wavre, Belgium; GSK, Wavre, Belgium

**Keywords:** respiratory syncytial virus, adjuvanted RSVPreF3, young adults, noninferior immunogenicity, safety

## Abstract

**Background:**

The AS01_E_-adjuvanted respiratory syncytial virus (RSV) prefusion F protein-based vaccine (adjuvanted RSVPreF3) is approved for ≥60-year-olds and 50–59-year-olds at increased risk of RSV disease. We evaluated the vaccine's immunogenicity and safety in 18–49-year-olds at increased risk of RSV disease due to certain chronic conditions.

**Methods:**

This open-label, multicountry phase 3b trial included 18–49-year-olds at increased risk (at-risk 18–49 group) and a control group of ≥60-year-olds with or without chronic conditions (≥60 group). Primary objective was to demonstrate immunological noninferiority to adjuvanted RSVPreF3 in the at-risk 18–49 versus ≥60 group, based on group RSV-A/RSV-B geometric mean titer ratios and seroresponse rate differences 1 month postvaccination. Humoral, cell-mediated immunogenicity, and safety were assessed until 6 months postvaccination.

**Results:**

Overall, 1458 adults were vaccinated. Immunological noninferiority was demonstrated in the at-risk 18–49 versus ≥60 group at 1 month postvaccination. Both groups showed increased RSV-A/RSV-B neutralizing titers and RSVPreF3-specific CD4+ T-cell frequencies at 1 month postvaccination that declined but remained above baseline at 6 months. Solicited events were more frequent in the at-risk 18–49 (84.3%) than in the ≥60 group (69.4%); most were mild-to-moderate and transient. Rates and intensity of unsolicited adverse events (AEs) were comparable across groups, with low reporting of serious AEs and AEs of special interest.

**Conclusions:**

Adjuvanted RSVPreF3 was immunologically noninferior in 18–49-year-olds with underlying conditions compared with ≥60-year-olds in whom efficacy was previously demonstrated, supporting efficacy inference in this younger population. Up to 6 months postvaccination, the vaccine elicited robust immune responses, and the safety profile was acceptable.

**Clinical Trial Registration**: NCT06389487

## Teaser

The AS01_E_-adjuvanted RSVPreF3 vaccine demonstrated immunological noninferiority in adults 18–49 years at increased risk of RSV disease due to certain chronic conditions versus adults ≥60 years, showing persistent immunogenicity until 6 months postvaccination with an acceptable safety profile.

Respiratory syncytial virus (RSV) poses a substantial burden to adults, causing illnesses that range from mild upper respiratory tract infections to severe, life-threatening RSV-related lower respiratory tract disease (RSV-LRTD) [1]. Up to 50% of infected adults develop RSV-LRTD [2–4], with increasing age [5–7] and immunocompromised status [8] as significant risk factors. Additionally, adults of all ages with certain chronic medical conditions, particularly pulmonary diseases like chronic obstructive pulmonary disease (COPD), asthma, and cystic fibrosis, as well as cardiovascular, liver, kidney, and metabolic diseases (including diabetes), are at increased risk of RSV. These conditions significantly predispose individuals to severe RSV-LRTD, often leading to complications like pneumonia or exacerbations of existing illnesses, which result in higher hospitalization and mortality rates [9–12].

The AS01_E_-adjuvanted RSV prefusion F protein-based vaccine (adjuvanted RSVPreF3, *Arexvy,* GSK), is licensed for use in adults ≥60 years of age (YOA) since 2023 [13, 14]. In the pivotal AReSVi-006 trial, the vaccine demonstrated 82.6% efficacy against RSV-LRTD and 94.1% against severe RSV-LRTD over 1 RSV season in adults ≥60 YOA with or without underlying chronic conditions that increase risk of RSV disease, with 94.6% efficacy against RSV-LRTD in those with chronic conditions [15]. Protection was sustained for at least 3 seasons (67.2% in season 2; 62.9% in season 3) [16, 17].

The vaccine also demonstrated a noninferior immune response and a comparable safety profile in adults 50–59 YOA with or without chronic conditions, compared with those ≥60 YOA [18], leading to its approval for use in adults 50–59 YOA at increased risk of RSV disease [13, 14].

The adjuvanted RSVPreF3 has not been evaluated in adults <50 YOA, including those with chronic conditions, but expanding its access to this younger population, particularly those at increased risk of RSV disease, could help reduce RSV-related diseases. This study aimed to demonstrate a noninferior humoral immune response to adjuvanted RSVPreF3 in adults 18–49 YOA at increased risk compared with those ≥60 YOA, in whom efficacy was previously demonstrated [15–17], using an immunobridging design [19, 20]. Humoral and cell-mediated immunogenicity (CMI) and the vaccine's safety profile were assessed until 6 months postvaccination.

**Figure 1.**
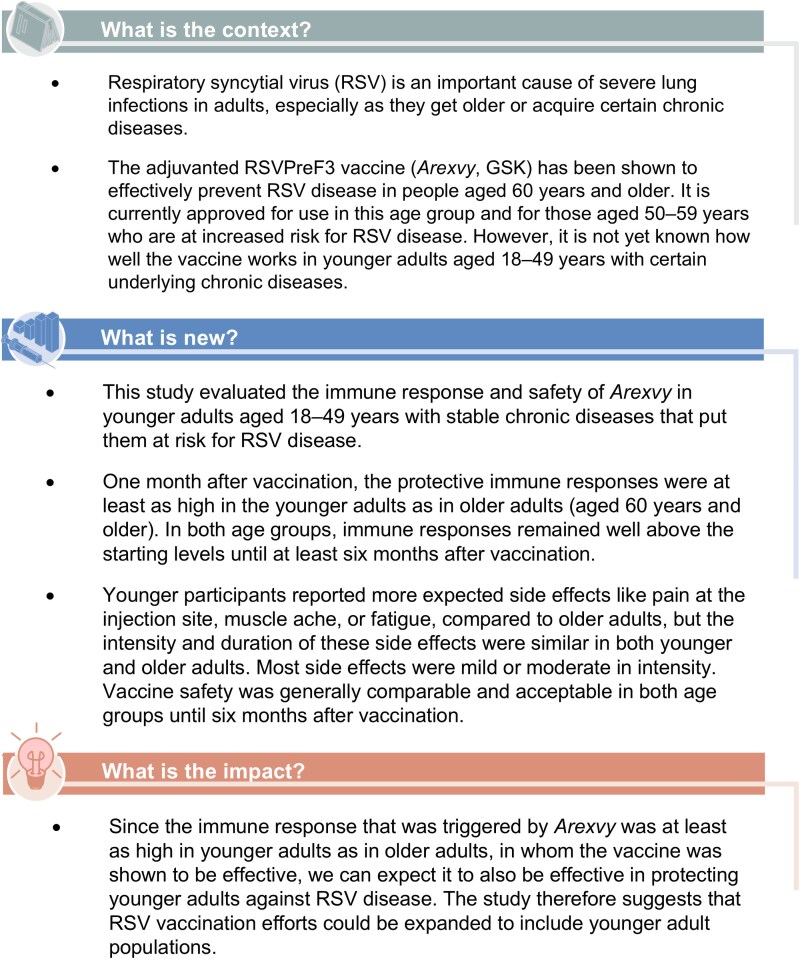
Plain language summary.

Figure 1 presents a summary of the study in plain language.

## METHODS

### Study Design, Participants, and Interventions

This phase 3b, open-label, nonrandomized study (NCT06389487) was conducted in 6 countries across 52 sites: Australia (7), Canada (12), Germany (9), Japan (4), South Africa (4), and the United States (16) (Supplementary Table 1). The study enrolled participants from 29 April 2024 to 16 September 2024 and was completed on 18 March 2025.

**Figure 2.**
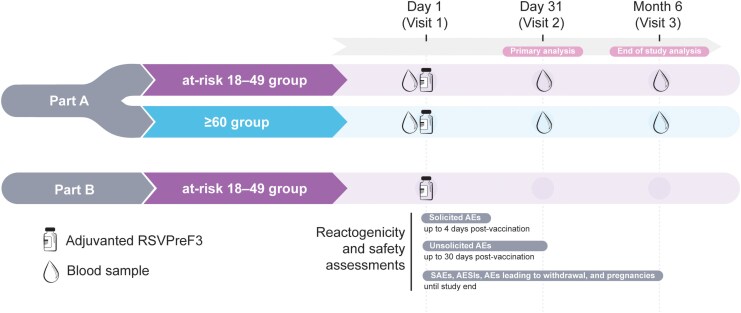
Study design. At-risk 18–49 group, group of participants aged 18–49 y at increased risk of respiratory syncytial virus (RSV) disease; ≥60 group, group of participants aged ≥60 y; adjuvanted RSVPreF3, AS01_E_-adjuvanted RSV prefusion F protein-based vaccine; AE, adverse event; SAE, serious AE; AESI, AE of special interest. Notes: An additional volume of blood was collected from a subset of participants in Part A of the study for cell-mediated immunogenicity assessment (CMI subset). The first participants of each group in Part A, if possible, were allocated to the CMI subset until the allocated target sample size was reached (see Supplementary materials). Participants in Part B had a phone contact at Month 6.

The study was conducted in 2 parts. Part A focused on immunogenicity and safety, and included 2 parallel cohorts: adults 18–49 YOA at increased risk (at-risk 18–49 group), and adults ≥60 YOA (≥60 group). Part B included an additional at-risk 18–49 cohort to better characterize the vaccine's safety profile in this population (Figure 2).

The at-risk 18–49 cohorts included nonimmunocompromised adults 18–49 YOA, diagnosed with at least one of the following medical conditions commonly associated with an increased risk of RSV disease: chronic pulmonary disease (COPD, asthma, cystic fibrosis, lung fibrosis, restrictive lung disease, interstitial lung disease, emphysema, and bronchiectasis), chronic cardiovascular disease (chronic heart failure, coronary artery disease, and cardiac arrhythmia), diabetes mellitus types 1/2, chronic kidney disease, chronic liver disease, and active or chronic neurological/neuromuscular conditions. All chronic conditions had to be considered stable by the investigator (ie, no significant changes in therapy or disease severity in the 3 months prior to enrollment). Eligibility criteria were applied to the severity of certain conditions to ensure a strong association with increased RSV risk (eg, activity-restricting symptoms or long-term medication).

The ≥60 group served as a control. The same eligibility criteria as in the AReSVi-006 efficacy study [15] were applied (ie, adults ≥60 YOA with or without stable chronic conditions).

Individuals with unstable illness, dementia, or a confirmed/suspected immunosuppressive or immunodeficiency condition due to disease or immunosuppressive/cytotoxic therapy, as well as those who had previously received any RSV vaccine or chronic/long-acting immune-modifying drugs 3–6 months before study vaccination were not eligible. Enrollment rules were applied to ensure representation by sex (all cohorts), disease category (at-risk 18–49 cohorts), and age (≥60 cohort) (Supplementary materials). All eligible participants received 1 dose of adjuvanted RSVPreF3 on Day 1 and were followed for 6 months. A protocol summary is available at https://www.gsk-studyregister.com/en/trial-details/?id=222253. Information regarding ethical conduct is available in the Supplementary materials.

### Objectives

The primary objective was to demonstrate noninferiority of the humoral immune response to adjuvanted RSVPreF3 in the at-risk 18–49 group compared with the ≥60 group, both in terms of group RSV-A/RSV-B adjusted geometric mean titer (GMT) ratios (GMRs) and group seroresponse rate (SRR) differences from baseline, at 1 month postvaccination.

Secondary objectives included further characterization of humoral immunogenicity, and evaluation of the vaccine's reactogenicity and safety in both study groups until 6 months postvaccination. Characterization of CMI was a tertiary objective.

### Immunogenicity Evaluation

Blood samples for humoral immunogenicity analyses were collected from participants in Part A of the study at baseline (Day 1, prevaccination), 1 month (Day 31), and 6 months postvaccination. RSV-A/RSV-B neutralizing titers were assessed using GSK neutralization assays and expressed in estimated dilution 60 [15].

For CMI analyses, an additional blood volume was collected from a selected number of participants in Part A at the same time points. Frequencies of RSVPreF3-specific CD4+/CD8+ T cells (per million CD4+/CD8+ T cells), expressing ≥2 activation markers—including ≥1 cytokine—among CD40 ligand, 4-1BB, interleukin-2, tumor necrosis factor-α, interferon-ɣ, interleukin-13, and interleukin-17, were determined by intracellular cytokine staining of peripheral blood mononuclear cells [21].

### Reactogenicity and Safety Evaluations

Solicited administration-site and systemic events starting within 4 days postvaccination were recorded by all participants using an electronic diary. Unsolicited adverse events (AEs) within 30 days postvaccination were recorded by the investigator in electronic case report forms. Serious AEs (SAEs), AEs leading to study discontinuation, and AEs of special interest (AESIs), including potential immune-mediated diseases (pIMDs) and atrial fibrillation, were recorded up to study end, or up to Day 30 in case of nonserious atrial fibrillation events. The investigator graded the intensity of AEs from mild (grade 1) to severe (grade 3) (Supplementary materials) and determined the causal relationship between the vaccine and each AE.

All AEs were followed until resolved, until study end, until the participant was lost to follow-up, or until SAEs and nonserious AESIs were considered stabilized.

Pregnancies were documented until study end and followed until 6–8 weeks after delivery.

### Statistical Methods

Primary and secondary humoral immunogenicity objectives were analyzed on the per-protocol set (PPS, all eligible participants who received the vaccine as per protocol, had immunogenicity results both prevaccination and postvaccination available, complied with blood draw intervals, had no interfering intercurrent conditions, and did not receive prohibited concomitant medications/vaccinations). CMI objectives were analyzed on the PPS for CMI subset.

The primary noninferiority objective was assessed according to a hierarchical testing procedure (first for RSV-A, and, if met, for RSV-B) to control the global 1-sided type I error at 2.5%. Noninferiority was evaluated both in terms of group adjusted GMR (≥60 group over at-risk 18–49 group) and group SRR difference (≥60 group minus at-risk 18–49 group) at 1 month postvaccination. SRR was defined as the proportion of participants with a ≥ 4-fold increase in neutralizing titers from baseline to the postvaccination time point. Group adjusted GMRs with 95% CIs were computed using an ANCOVA model on log_10_-transformed titers, with group and baseline log_10_-transformed titers as covariates; group SRR differences with 95% CIs were computed using the Miettinen and Nurminen method [22]. Noninferiority was demonstrated if the upper limit of the 2-sided 95% confidence interval (CI) was ≤1.5 for the group GMR and ≤10% for the group SRR difference.

RSV-A and RSV-B neutralizing GMTs and mean geometric increases (MGIs) (ie, geometric mean of the within-participant ratios 1 month or 6 months postvaccination over baseline) were calculated with 95% CIs. Titers below the lower limit of quantification (LLOQ) were replaced by half the LLOQ and titers above the upper limit of quantification (ULOQ) by the ULOQ. Missing data were not replaced. RSVPreF3-specific CD4+/CD8+ T-cell frequencies (per million CD4+/CD8+ T cells) were summarized using descriptive statistics.

Demographic characteristics, reactogenicity, and safety were analyzed on all vaccinated participants and summarized using descriptive statistics. For these safety analyses, data from all at-risk participants 18–49 YOA in study Parts A and B were pooled.

Details regarding sample size calculations are provided in Supplementary materials.

Statistical analyses were performed using SAS (SAS Institute Inc., Cary, North Carolina, US).

## RESULTS

### Study Participants

**Figure 3.**
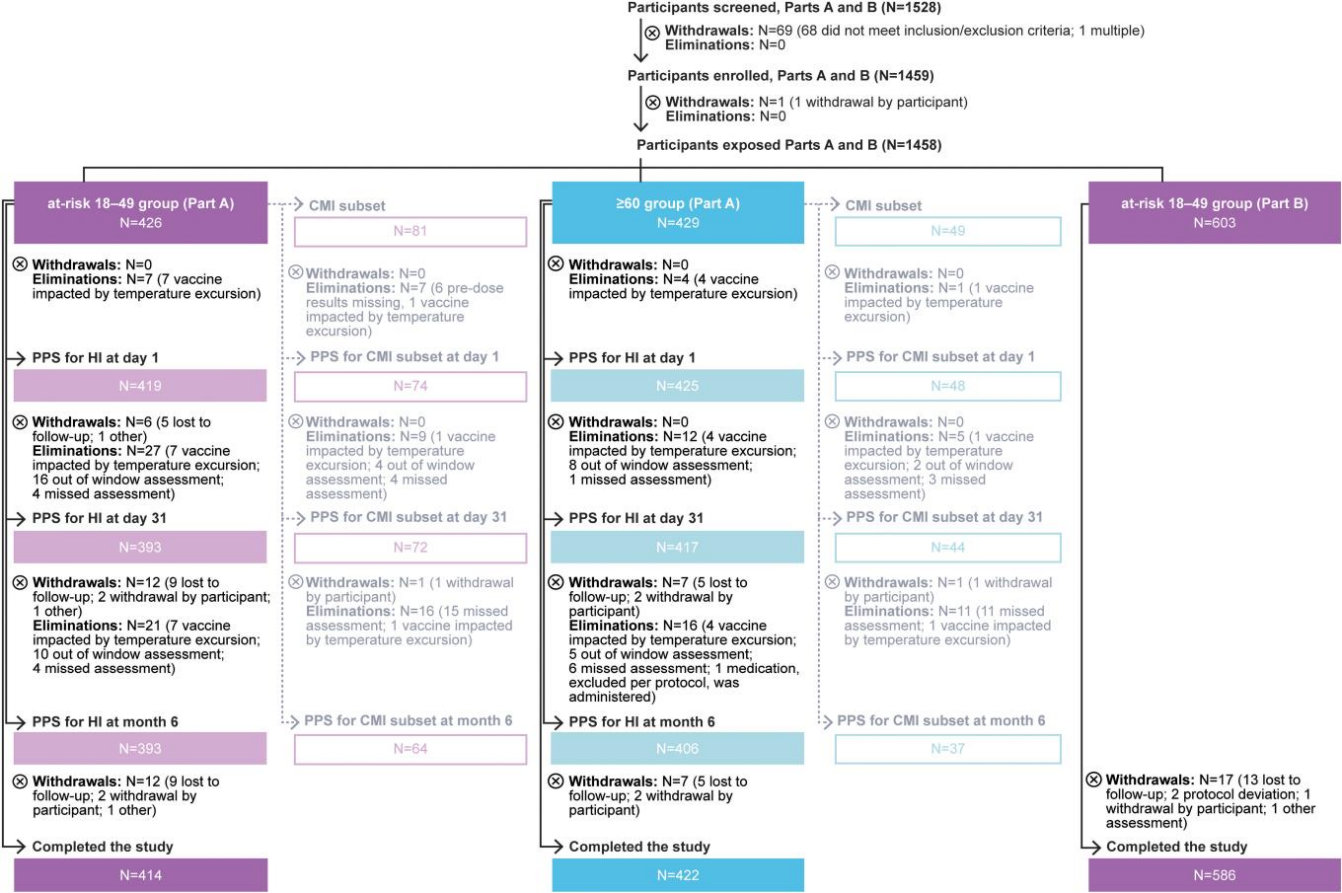
Participant flow. N, number of participants; at-risk 18–49 group, group of participants aged 18–49 y at increased risk of respiratory syncytial virus disease; ≥60 group, group of participants aged ≥60 y; PPS, per-protocol set; HI, humoral immunogenicity; CMI, cell-mediated immunogenicity. Note: For eliminations, multiple reasons could apply for 1 participant; the figure lists all reasons.

In total, 1459 participants were enrolled, and 1458 (at-risk 18–49 group: 1029; ≥60 group: 429) received adjuvanted RSVPreF3 on Day 1 (Figure 3). Overall, 1445 participants (99.1%) completed visit 2 (Day 31), and 1422 (97.5%) completed visit 3 (Month 6). Thirty-six participants (2.5%) were withdrawn by study end, mainly due to loss to follow-up (27 participants). None withdrew due to an AE.

Of the 855 participants in Part A, 844 were included in the PPS for humoral immunogenicity at Day 1, 810 at Day 31, and 799 at Month 6. The main reasons for exclusion were immunogenicity assessment outside the protocol window (24 participants at Day 31; 15 at Month 6) or administration of vaccine impacted by a temperature excursion (11 participants at each time point). The PPS for CMI subset consisted of 122 participants at Day 1, 116 at Day 31, and 101 at Month 6.

Overall, the demographic and baseline characteristics (excluding age) of the vaccinated participants were comparable between the at-risk 18–49 and ≥60 groups (Table 1). The mean age was 38.4 years in the at-risk 18–49 and 68.6 years in the ≥60 group. In the at-risk 18–49 group, 68.8% of participants had 1 chronic condition and the remainder had ≥2. In the ≥60 group, 34.8% had no condition, 35.4% had 1 condition, and 29.8% had ≥2. The most common conditions were cardiopulmonary diseases (55.0% in the at-risk 18–49 and 31.5% in the ≥60 group) and diabetes mellitus (48.9% and 30.3%, respectively) (Table 1).

**Table 1.** Demographic and Baseline Characteristics of Study Participants, All Vaccinated Participants

Characteristic	At-risk 18–49 Group N = 1029	≥60 Group N = 429
Mean (SD) age at vaccination, y	38.4 (8.4)	68.6 (5.7)
Age category, n (%)		
18–49 y	1029 (100)	…
60–69 y	…	248 (57.8)
≥70 y	…	181 (42.2)
≥80 y	…	18 (4.2)
Female sex, n (%)	594 (57.7)	220 (51.3)
Race, n (%)		
Asian	124 (12.1)	50 (11.7)
Black/African American	258 (25.1)	54 (12.6)
White	529 (51.4)	302 (70.4)
Other races^a^	118 (11.5)	23 (5.4)
Ethnicity		
Not Hispanic or Latino	892 (86.7)	398 (92.8)
Other ethnicities^b^	137 (13.3)	31 (7.2)
Mean (SD) BMI, kg/m^2^ ^c^	31.4 (8.7)	29.0 (6.2)
Smoking status for tobacco		
Current smoker	211 (20.5)	55 (12.8)
Former smoker	186 (18.1)	166 (38.7)
Never smoker	632 (61.4)	208 (48.5)
Smoking status for e-cigarettes		
Current smoker	53 (5.2)	6 (1.4)
Former smoker	38 (3.7)	5 (1.2)
Never smoker	937 (91.1)	417 (97.2)
Unknown	1 (0.1)	1 (0.2)
Chronic disease of interest^d^		
One chronic disease of interest	708 (68.8)	152 (35.4)
≥Two chronic diseases of interest	319 (31.0)	128 (29.8)
Cardiopulmonary conditions	566 (55.0)	135 (31.5)
Diabetes mellitus	503 (48.9)	130 (30.3)
Other chronic diseases of interest	276 (26.8)	129 (30.1)

At-risk 18–49 group, group of participants aged 18–49 y at increased risk of respiratory syncytial virus (RSV) disease; ≥60 group, group of participants aged ≥60 y; N, number of participants who received the vaccine; SD, standard deviation; y, years; n (%), number (percentage) of participants in a given category; BMI, body mass index.

^a^Includes American Indian/Alaska Native, Native Hawaiian/other Pacific Islander, multiple race categories, race not reported, or race unknown.

^b^Includes Hispanic or Latino, ethnicity not reported, or ethnicity unknown.

^c^BMI data were missing for 1 participant in the at-risk 18–49 group.

^d^Chronic conditions of interest refer to the predefined conditions that are known to increase the risk of RSV disease: chronic obstructive pulmonary disease (Global Initiative for Chronic Obstructive Lung Disease grade 2–4), asthma (on maintenance and reliever therapy or frequent rescue treatment), cystic fibrosis, lung fibrosis, restrictive lung disease, interstitial lung disease, emphysema, bronchiectasis, chronic heart failure (New York Heart Association class II or higher), coronary artery disease, cardiac arrhythmia (requiring medical support), diabetes mellitus types 1 and 2 (with active treatment for at least the past 6 m), chronic kidney disease (G2-G3), chronic liver disease (moderate to severe), and active or chronic neurological/neuromuscular conditions.

The demographic and baseline characteristics of the PPS were generally similar to all vaccinated participants; some differences were noted in the PPS for CMI subset (Supplementary tables 2 and 3).

### Immunogenicity

#### Immunological Noninferiority

**Figure 4.**
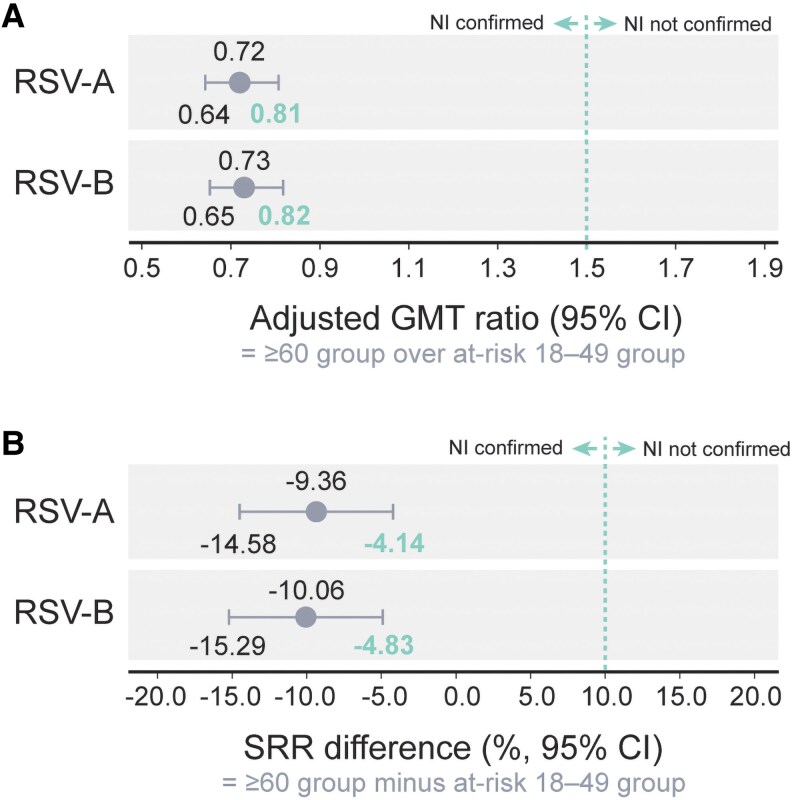
Noninferiority of the humoral immune response to adjuvanted RSVPreF3 in terms of group (*A*) adjusted geometric mean titer ratios and (*B*) seroresponse rate differences of RSV-A and RSV-B neutralizing titers in adults aged 18–49 y at increased risk of RSV disease compared with adults ≥60 y at 1 m postvaccination, per-protocol set (primary analysis). Adjuvanted RSVPreF3, AS01_E_-adjuvanted respiratory syncytial virus (RSV) prefusion F protein-based vaccine; NI, noninferiority; adjusted GMT ratio, adjusted GMT of the ≥60 group over adjusted GMT of the at-risk 18–49 group; SRR, seroresponse rate, ie, proportion of participants with a ≥4-fold increase in neutralizing titers over baseline at 1 m postvaccination; SRR difference, seroresponse rate of the ≥60 group minus the seroresponse rate of the at-risk 18–49 group; CI, confidence interval; at-risk 18–49 group, group of participants aged 18–49 y at increased risk of RSV disease; ≥60 group, group of participants aged ≥60 y. Notes: Noninferiority was demonstrated if the upper limit of the 2-sided 95% CI was ≤1.5 for the group adjusted GMT and ≤10% for the group SRR difference. Noninferiority margins are indicated with a dashed line on the graphs. For RSV-A, 394 participants in the at-risk 18–49 group and 417 in the ≥60 group had both prevaccination and postvaccination data available to determine the group GMR. For RSV-B, these numbers were 393 and 417, respectively. For the determination of the group SRR difference, 343 participants in the at-risk 18–49 group and 324 in the ≥60 group had a ≥4-fold increase in neutralizing titers over baseline at 1 m postvaccination for RSV-A, while for RSV-B, the numbers were 343 in the at-risk 18–49 group and 322 in the ≥60 group.

One month postvaccination, the primary analysis demonstrated a noninferior humoral immune response in the at-risk 18–49 compared with the ≥60 group. The group GMRs were 0.72 (95% CI: 0.64, .81) for RSV-A and 0.73 (0.65, 0.82) for RSV-B. The group SRR differences were −9.36% (−14.58, −4.14) for RSV-A and −10.06% (−15.29, −4.83) for RSV-B. Hence, the upper limits of the 2-sided 95% CIs for the group GMRs and group SRR differences were below their predefined noninferiority margins of 1.5% and 10%, respectively, for both RSV-A and RSV-B (Figure 4).

#### Humoral Immunogenicity

**Figure 5.**
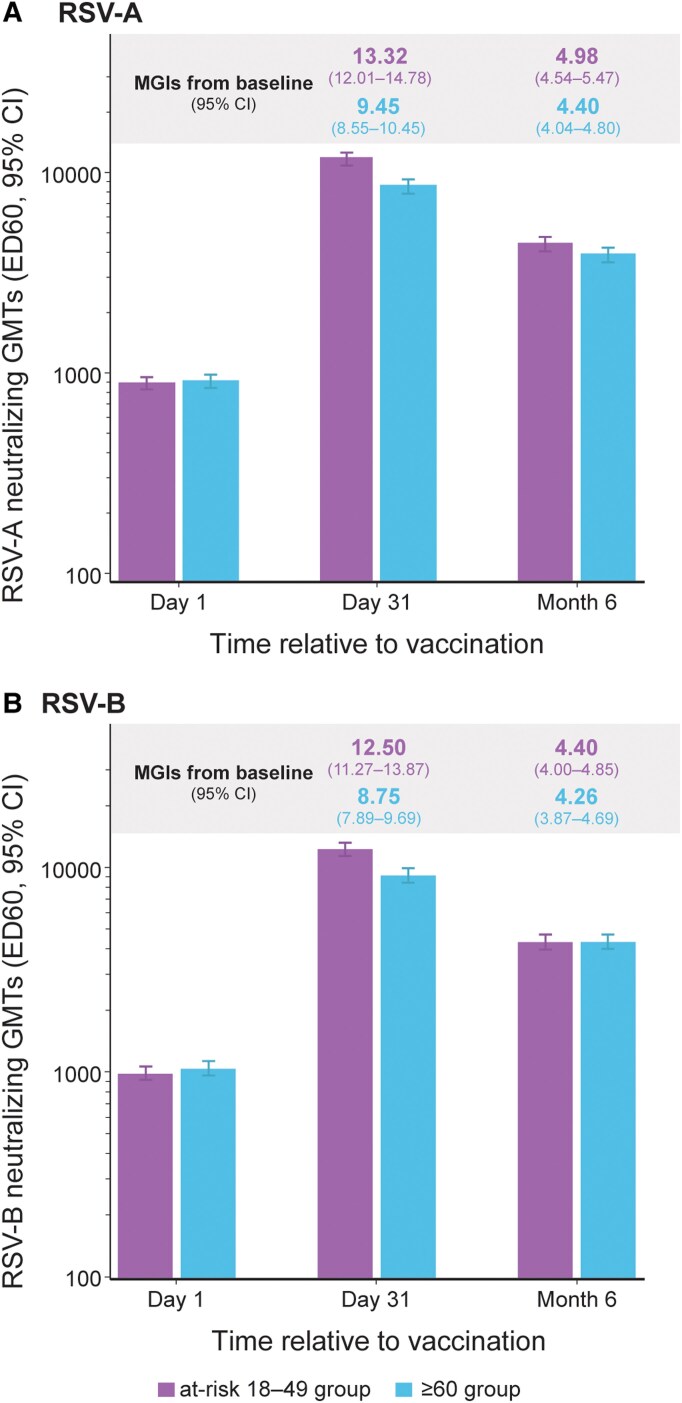
*A*, RSV-A and *B,* RSV-B neutralizing titers, per-protocol set (end of study analysis). GMT, geometric mean titer; ED60, estimated dilution 60; CI, confidence interval; MGI, mean geometric increase; at-risk 18–49 group, group of participants aged 18–49 y at increased risk of respiratory syncytial virus (RSV) disease; ≥60 group, group of participants aged ≥60 y. Note: The values on top of the relevant bars at Day 31 and Month 6 indicate the MGIs compared with baseline. For each variable (GMT or MGI), the number of participants per group and per time point with available data can be found in Table 2.

One month postvaccination, RSV-A and RSV-B neutralizing titers increased in both the at-risk 18–49 and ≥60 groups compared with baseline (Figure 5 and Table 2). For RSV-A, MGIs over baseline were 13.32 in the at-risk 18–49 and 9.45 in the ≥60 group; for RSV-B, MGIs were 12.50 and 8.75, respectively. For RSV-A, SRRs were 87.0% in the at-risk 18–49 and 77.7% in the ≥60 group; for RSV-B, SRRs were 87.2% and 77.2%, respectively (Table 2).

**Table 2.** RSV-A and RSV-B Neutralizing Titers, per-protocol Set (End of Study Analysis)

		At-risk 18–49 Group		≥60 Group	
Time point	Variable	N	Value or % (95% CI)	N	Value or % (95% CI)
RSV-A neutralizing titer					
Baseline	GMT	419	892.6 (821.5–969.9)	425	913.8 (837.2–997.4)
Day 31	GMT	393	11 853.4 (10 875.3–12 919.5)	417	8632.4 (7858.3–9482.6)
	MGI	393	13.32 (12.01–14.78)	417	9.45 (8.55–10.45)
	SRR	342	87.0 (83.3–90.2)	324	77.7 (73.4–81.6)
Month 6	GMT	393	4439.8 (4042.1–4876.7)	406	3917.7 (3553.0–4319.7)
	MGI	393	4.98 (4.54–5.47)	406	4.40 (4.04–4.80)
	SRR	242	61.6 (56.6–66.4)	207	51.0 (46.0–55.9)
RSV-B neutralizing titer					
Baseline	GMT	418	988.1 (905.9–1077.8)	425	1045.1 (953.7–1145.2)
Day 31	GMT	393	12 337.6 (11 334.3–13 429.7)	417	9178.5 (8367.7–10 067.8)
	MGI	392	12.50 (11.27–13.87)	417	8.75 (7.89–9.69)
	SRR	342	87.2 (83.5–90.4)	322	77.2 (72.9–81.2)
Month 6	GMT	393	4335.6 (3941.7–4769.0)	406	4355.0 (3971.4–4775.7)
	MGI	392	4.40 (4.00–4.85)	406	4.26 (3.87–4.69)
	SRR	204	52.0 (47.0–57.1)	201	49.5 (44.5–54.5)

At-risk 18–49 group, group of participants aged 18–49 y at increased risk of respiratory syncytial virus (RSV) disease; ≥60 group, group of participants aged ≥60 y; GMT, geometric mean titer; MGI, mean geometric increase; SRR, seroresponse rate, ie, proportion of participants with a ≥4-fold increase in neutralizing titers from baseline to the respective postvaccination time point; N for GMT, number of participants with available results at given time point; N for MGI, number of participants with available results at both the given time point and at baseline; N for SRR, number of participants with a ≥4-fold increase in neutralizing titers from baseline to the respective postvaccination time point; CI, confidence interval.

Six months postvaccination, RSV-A and RSV-B neutralizing titers declined in both groups compared with 1 month postvaccination but remained above baseline (Figure 5 and Table 2). For RSV-A, MGIs over baseline were 4.98 in the at-risk 18–49 and 4.40 in the ≥60 group; for RSV-B, MGIs were 4.40 and 4.26, respectively. SRRs were 61.6% and 51.0% for RSV-A, and 52.0% and 49.5% for RSV-B, respectively (Table 2).

#### Cell-mediated Immunogenicity

**Figure 6.**
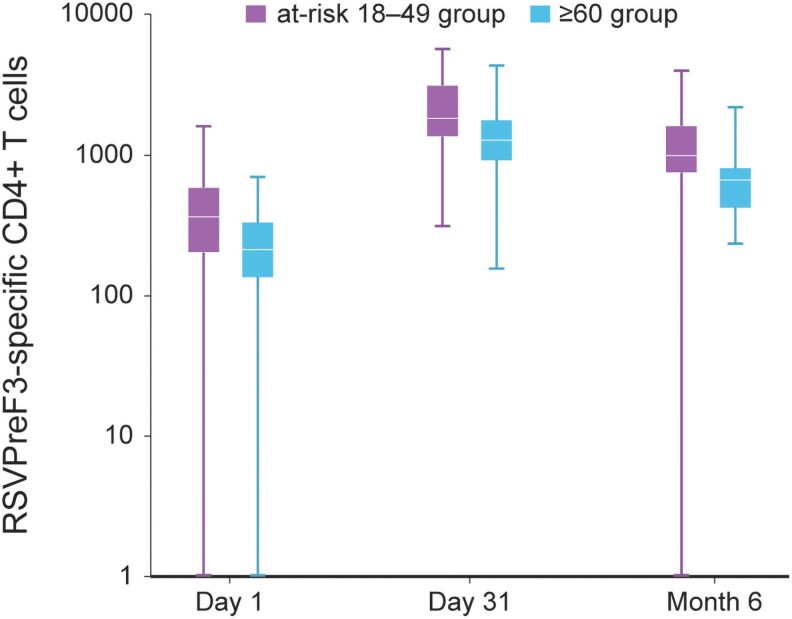
Frequency of RSVPreF3-specific CD4+ T cells (per million CD4+ T cells) expressing at least 2 activation markers, including at least 1 cytokine among CD40L, 4-1BB, IL-2, TNF-α, IFN-γ, IL-13, and IL-17, per-protocol set for CMI subset (end of study analysis). RSVPreF3, respiratory syncytial virus (RSV) prefusion F protein 3; CD40L, CD40 ligand; IL, interleukin; TNF-α, tumor necrosis factor-α; IFN-γ, interferon-γ; CMI, cell-mediated immunogenicity; at-risk 18–49 group, group of participants aged 18–49 y at increased risk of RSV disease; ≥60 group, group of participants aged ≥60 y. Notes: The boxplots display the minimum, first quartile (Q1), median, third quartile (Q3), and maximum values. At baseline (Day 1), CMI results were available for 69 participants in the at-risk 18–49 group and 48 in the ≥60 group. At Day 31, these numbers were 66 and 43, respectively, and at Month 6, they were 62 and 37, respectively.

At baseline, the median frequency of RSVPreF3-specific CD4+ T cells expressing ≥2 activation markers (per million CD4+ T cells) was 366.0 in the at-risk 18–49 and 213.0 in the ≥60 group. One month postvaccination, frequencies increased to 1834.5 in the at-risk 18–49 and to 1288.0 in the ≥60 group, and then declined at Month 6 to 997.0 in the at-risk 18–49 and to 667.0 in the ≥60 group (Figure 6). No RSVPreF3-specific CD8+ T-cell response was observed.

### Reactogenicity and Safety

**Figure 7.**
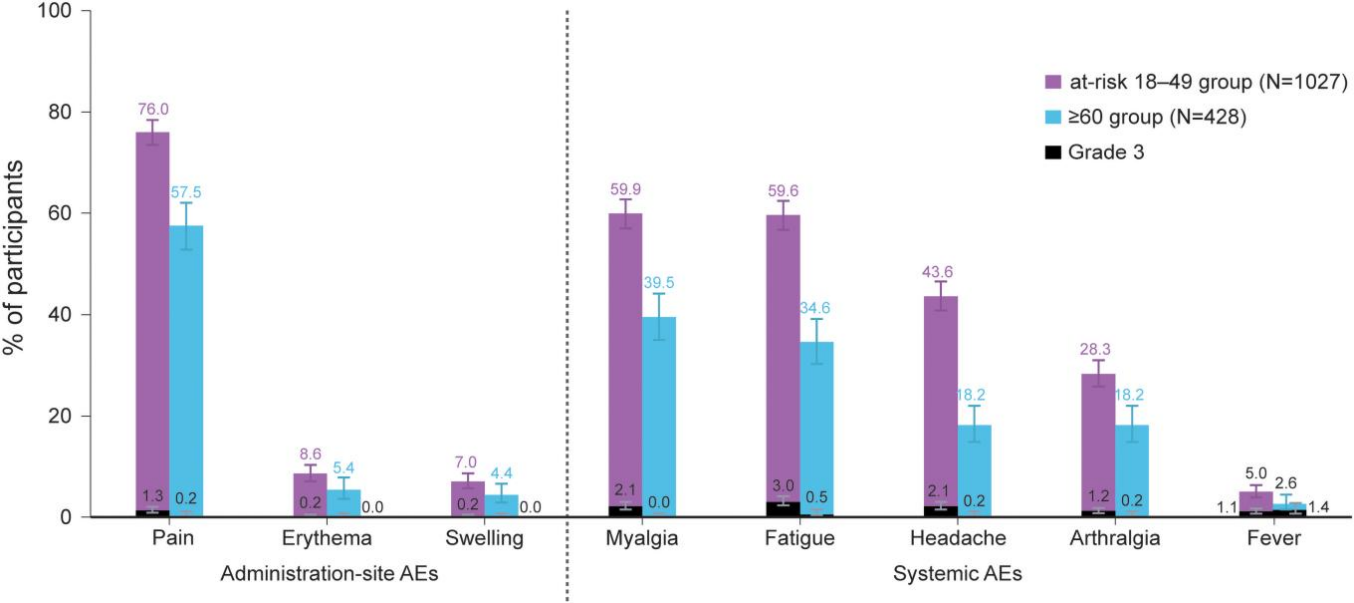
Solicited events with onset within 4 d postvaccination, all vaccinated participants with available results. At-risk 18–49 group, group of participants aged 18–49 y at increased risk of respiratory syncytial virus disease; N, number of participants; ≥60 group, group of participants aged ≥60 y; AE, adverse event; Grade 3 solicited AEs, solicited AEs that prevent normal everyday activities, fever with a temperature >39.0°C, or erythema/swelling with a surface diameter >100 mm.

Within 4 days postvaccination, 84.3% of participants in the at-risk 18–49 group and 69.4% in the ≥60 group reported ≥1 solicited event. Solicited administration-site events were reported by 76.4% of participants in the at-risk 18–49 and 58.2% in the ≥60 group, with injection site pain being the most frequent (76.0% and 57.5%, respectively) (Figure 7). Grade 3 solicited administration-site events were reported by 1.6% and 0.2% of participants, respectively. Solicited systemic events were reported by 75.0% in the at-risk 18–49 group and 54.9% in the ≥60 group, with myalgia (59.9% and 39.5%) and fatigue (59.6% and 34.6%) being the most frequent. Grade 3 solicited systemic events occurred in 5.8% and 2.3% of participants, respectively. Most solicited events were mild or moderate in intensity and resolved within 3 days in both groups.

Unsolicited AEs within 30 days postvaccination were reported by 18.5% of participants in the at-risk 18–49 and 17.7% in the ≥60 group; 8.7% and 3.0%, respectively, experienced an unsolicited AE that was considered vaccine-related by the investigator (Table 3). Grade 3 unsolicited AEs were reported in 0.5% and 1.2% of participants, respectively; none were considered vaccine-related. Up to study end, 1.4% of participants in the at-risk 18–49 and 3.0% in the ≥60 group reported ≥1 SAE; none were considered vaccine-related. No fatal outcomes were reported.

**Table 3.** Unsolicited Adverse Events, Serious Adverse Events, and Adverse Events of Special Interest, All Vaccinated Participants

	At-risk 18–49 Group N = 1029	≥60 Group N = 429
Event	n (%)	n (%)
Unsolicited AEs within 30 d following vaccination		
Any grade unsolicited AE	190 (18.5)	76 (17.7)
Any grade unsolicited AE considered related to vaccine^a^	90 (8.7)	13 (3.0)
Grade 3 unsolicited AE	5 (0.5)	5 (1.2)
SAEs up to study end (6 m)		
Any SAE	14 (1.4)	13 (3.0)
SAE considered related to vaccine^a^	0	0
Fatal SAE	0	0
AEs of special interest up to study end (6 m)		
Any pIMD	2 (0.2)	1 (0.2)
pIMD considered related to vaccine^a^	1 (0.1)	0
Any atrial fibrillation^b,c^	1	1
Atrial fibrillation considered related to vaccine^a^	0	0
Any AE leading to study discontinuation	0	0
Pregnancy^a,b^	3	0

At-risk 18–49 group, group of participants aged 18–49 y at increased risk of respiratory syncytial virus disease; ≥60 group, group of participants aged ≥60 y; N, number of participants who received the vaccine; n (%), number (percentage) of participants presenting ≥1 type of adverse event (AE) in a given category; SAE, serious AE; pIMD, potential immune-mediated disease.

^a^By investigator assessment.

^b^Only n is reported for atrial fibrillation and pregnancy events.

^c^Nonserious atrial fibrillation events were recorded within 30 d postvaccination.

Two pIMDs were reported in the at-risk 18–49 group and 1 in the ≥60 group (Table 3; Supplementary Table 4). One case in the at-risk 18–49 group was considered vaccine-related by the investigator; it involved an exacerbated hematoma (onset 14 days postvaccination; nonserious and mild in intensity), that initially developed at an insulin injection site, with other lesions appearing at other body parts over time. The case was classified as a pIMD by the investigator; clinical and laboratory evidence did not suggest an autoimmune etiology. The case resolved by itself 217 days later. One participant from each group reported atrial fibrillation; both occurred in participants with a medical or family history of atrial fibrillation/arrhythmia and were not considered vaccine-related.

During the study, 3 pregnancies were reported: 1 full-term pregnancy before and 2 after the last study visit, all of which resulted in live infants with no apparent congenital anomalies. None of these participants reported SAEs or pIMDs.

## DISCUSSION

Expanding access for adjuvanted RSVPreF3 to adults <50 YOA, particularly those at increased risk of RSV disease, could play a key role in further reducing RSV-related diseases. In this study, adjuvanted RSVPreF3 induced a noninferior humoral immune response in adults 18–49 YOA with chronic conditions that put them at increased risk of RSV disease compared with adults ≥60 YOA, in whom efficacy was previously demonstrated [15–17], supporting the inference of vaccine efficacy in this younger population. This inference is based on immunobridging, a well-established methodology [19] that follows World Health Organization guidelines for predicting vaccine efficacy in populations not included in efficacy trials using immunogenicity data in the absence of a correlate of protection [20]. The inclusion of the ≥60 group as a control group with eligibility criteria aligned with the efficacy study [15] enhances the validity of our findings.

The robust immune response observed in both age groups sustained for at least 6 months postvaccination. Both populations exhibited a similar trend over time in their RSV-A and RSV-B neutralizing titers, characterized by a sharp increase at 1 month postvaccination, followed by declining titers that remained above baseline levels until at least 6 months postvaccination; MGIs for the ≥60 group were consistent with previous observations in this population [23]. Although our study was designed to assess noninferiority rather than to compare responses between groups over time, a trend toward higher humoral immunogenicity was noted in participants 18–49 YOA compared with those ≥60 YOA at 1 month postvaccination, with responses converging at 6 months. In terms of CMI, frequencies of RSVPreF3-specific CD4+ T cells expressing ≥2 activation markers increased at 1 month postvaccination compared with baseline in both age groups, which then decreased but remained above baseline levels at 6 months postvaccination. Overall, the immunogenicity data suggest that adults 18–49 YOA may achieve at least similar levels of protection [15–17] and immune persistence [23] by adjuvanted RSVPreF3 compared with those ≥60 YOA.

While reactogenicity events were reported more frequently in the at-risk 18–49 group than in the ≥60 group, their duration and intensity were similar across groups. This difference is not unexpected and may be attributed to the generally lower inflammatory response in adults ≥60 YOA following vaccination [24], as also noted with other vaccines (eg, COVID-19 vaccines [25]).

Unsolicited AE, SAE, and AESI reporting rates were balanced between both populations. There was no pattern or cluster of events in any study group. No fatal outcomes were reported, and no SAEs were considered vaccine-related by the investigator. One pIMD considered vaccine-related in the at-risk 18–49 group and 1 atrial fibrillation event in each group (both not considered vaccine-related) were reported. Overall, the vaccine demonstrated an acceptable safety profile over 6 months in the at-risk 18–49 and ≥60 groups, consistent with previous observations in adults ≥60 YOA [15–17].

A key study strength is the demonstration of immunological noninferiority both in terms of the group GMRs and SRR differences of RSV-A/RSV-B neutralizing titers. While there is no established correlate of protection for RSV, serum neutralizing antibodies specific to the prefusion state of the RSV F protein are considered a key component of the immune response to reduce the risk of reinfection and severe disease in adults [26–29]. In adults ≥60 YOA, high serum neutralizing antibody levels have been associated with reduced hospitalization risk [30], and in pediatric populations, monoclonal neutralizing antibodies have been proven effective in lowering hospitalization rates and mortality [31–33].

One study limitation is the exclusion of immunocompromised individuals. This population is being studied in another phase 2b trial involving individuals ≥18 YOA who received a lung or kidney transplant.

Overall, the findings of this study indicate a favorable benefit-risk profile for the vaccine in adults 18–49 YOA, highlighting its potential to provide meaningful protection against RSV in younger adults.

## Supplementary Material

ciag058_Supplementary_Data

## Supplementary Data

Supplementary materials are available at *Clinical Infectious Diseases* online. Consisting of data provided by the authors to benefit the reader, the posted materials are not copyedited and are the sole responsibility of the authors, so questions or comments should be addressed to the corresponding author.
