# Supplementary material for: Immunogenicity and Safety of the AS01E-adjuvanted Respiratory Syncytial Virus (RSV) Prefusion F Protein Vaccine in Adults Aged 18–49 Years at Increased Risk of RSV Disease Compared with Adults Aged ≥60 Years
Source: Clin Infect Dis. 2026 Feb 27;82(5):e1093–103. doi: 10.1093/cid/ciag058 (PMC13189657; doi:10.1093/cid/ciag058)
Supplement: ciag058_Supplementary_Data [file ciag058_supplementary_data.docx]

**Supplementary materials**

**Immunogenicity and safety of the AS01_E_-adjuvanted respiratory syncytial virus (RSV) prefusion F protein vaccine in adults aged 18–49 years at increased risk of RSV disease compared to adults aged ≥60 years**

**Authors:** Essack Mitha^1^, Murdo Ferguson^2^, Agatha Cathrine Wilhase^3^, Ferdinandus De Looze^4^, Marie-Louise Vachon^5^, Helen Stacey^6^, Roy Rasalam^7,8^, Minoru Nozaki^9^, William B. Smith^10^, Tino F. Schwarz^11^, Hiwot Amare Hailemariam^12^, Quentin Deraedt^12^, Catherine Gérard^13^, Carline Vanden Abeele^12^, Silvia Damaso^12^, Dominique Descamps^12^, Judith Hill^12^, Dileep Dasyam^12^, Jonathan Van Gucht^12^, Veronica Hulstrøm^12^

**Affiliations:**

^1^Newtown Clinical Research Centre, Johannesburg, South Africa

^2^Colchester Research Group, Truro, Canada

^3^REIMED Pty., Boksburg, South Africa

^4^Momentum Clinical Research Wellers Hill, Brisbane, Australia

^5^Centre de Recherche du Centre Hospitalier Universitaire de Québec-Université Laval, Québec, Canada

^6^Diablo Clinical Research, Walnut Creek, California, United States

^7^Momentum Clinical Research Sunshine, Melbourne, Australia

^8^University of Melbourne, Melbourne, Australia

^9^Shirayurikai Swing Nozaki Clinic, Tokyo, Japan

^10^AMR Clinical, Knoxville, Tennessee, United States

^11^Institute of Laboratory Medicine and Vaccination Centre, Klinikum Würzburg Mitte, Juliusspital, Würzburg, Germany

^12^GSK, Wavre, Belgium

^13^GSK, Rixensart, Belgium

**Corresponding authors:**

Veronica Hulstrøm

GSK, Wavre, Belgium

Email: Veronica.x.hulstrom@gsk.com

Jonathan Van Gucht

GSK, Wavre, Belgium

Email: [Jonathan.x.vangucht@gsk.com](mailto:Jonathan.x.vangucht@gsk.com)

**Supplementary methods**

***Enrollment rules***

Enrollment rules were implemented to ensure adequate representation by sex, age, and underlying medical conditions across cohorts. In all study groups (from both Part A and B), approximately 35% of participants needed to be male and 35% were female, while the remaining 30% could be distributed freely across sex categories. In the at-risk 18–49 group (adults 18–49 years of age [YOA] at increased risk of respiratory syncytial virus [RSV] disease due to certain chronic medical conditions) from both Part A and B of the study, at least 25% of participants were required to have cardiopulmonary conditions, and at least 25% to have diabetes mellitus. The remaining 50% could include participants with either of these conditions or with chronic kidney or liver disease, or neurological or neuromuscular disorders. In the ≥60 group (including adults ≥60 YOA with or without certain stable chronic medical conditions), approximately 40% of participants were to be 60–69 YOA and 30% ≥70 YOA, with the remaining 30% distributed freely across these age categories.

***Ethical conduct***

The study was conducted in accordance with the protocol (summary available at <https://www.gsk-studyregister.com/en/trial-details/?id=222253>) and ethical principles derived from the Declaration of Helsinki, the Council for International Organizations of Medical Sciences guidelines, the International Council for Harmonization Good Clinical Practice guidelines, and all applicable laws and regulations. The study documents were approved by study center independent ethics committees or institutional review boards, and by the relevant local regulatory authorities, as applicable. Participants provided written or witnessed informed consent prior to performance of any study-specific procedures.

***Intensity grading of adverse events***

| **Event** | **Grade** | **Parameter** | |
| --- | --- | --- | --- |
| **Solicited administration-site events** | | |  |
| Pain | 1 | Mild: any pain neither interfering with nor preventing normal everyday activities | |
|  | 2 | Moderate: painful when limb is moved and interferes with normal everyday activities | |
|  | 3 | Severe: significant pain at rest that prevents normal everyday activities | |
| Erythema/swelling | 1 | Mild: greatest surface diameter >20 – ≤50 mm | |
|  | 2 | Moderate: greatest surface diameter >50 – ≤100 mm | |
|  | 3 | Severe: greatest surface diameter >100 mm | |
| **Solicited systemic events** | | | |
| Fever | 1 | Mild: temperature ≥38.0°C (100.4°F) – ≤38.5°C (101.3°F) | |
|  | 2 | Moderate: temperature >38.5°C (101.3°F) – ≤39.0°C (102.2°F) | |
|  | 3 | Severe: temperature >39.0°C (102.2°F) | |
| Headache/fatigue | 1 | Mild: easily tolerated | |
|  | 2 | Moderate: interferes with normal everyday activities | |
|  | 3 | Severe: prevents normal everyday activities | |
| Myalgia/arthralgia | 1 | Mild: present but does not interfere with normal everyday activities | |
|  | 2 | Moderate: interferes with normal everyday activities | |
|  | 3 | Severe: prevents normal everyday activities | |
| **Unsolicited AE/SAE/AESI** |  |  | |
| Any | 1 | Mild: usually transient and may require only minimal treatment or therapeutic intervention. The event does not generally interfere with normal everyday activities | |
|  | 2 | Moderate: usually alleviated with additional specific therapeutic intervention. The event interferes with normal everyday activities, causing discomfort but poses no significant or permanent risk of harm to the research participant | |
|  | 3 | Severe: interrupts normal everyday activities, or significantly affects clinical status, or may require intensive therapeutic intervention | |

AE, adverse event; SAE, serious AE; AESI, AE of special interest.

***Sample size calculations***

Assuming a 15% non-evaluable rate, a target of approximately 850 enrolled participants was set in Part A (425 participants each for both cohorts) to provide 361 evaluable participants per cohort for humoral immunogenicity analyses at 1-month post-vaccination. With this sample size and taking into account a hierarchical testing procedure to control the global type I error at 2.5% (1-sided), the power to demonstrate non-inferiority both in terms of adjusted geometric mean titer ratio and seroresponse rate (SRR) difference was 93% when assessed for RSV-A, and if demonstrated, the power was 90% when assessed for RSV-B. For reactogenicity and safety evaluation, the sample size of 1025 participants in the combined at-risk 18–49 cohorts of Part A and B of the study had 64%, 87%, and 95% probability of observing at least one vaccinated participant with an AE if the true incidence was 0.1%, 0.2%, or ≥0.3%, respectively.

For cell-mediated immunogenicity analysis, sample sizes of 75 participants were allocated to the at-risk 18–49 cohort and 50 participants to the ≥60 cohort.

**Supplementary results**

**Supplementary Table 1. Study sites**

| **Country** | **Site** |
| --- | --- |
| Australia | Sydney, NSW, Australia, 2065 |
|  | Tarragindi, QLD, Australia, 4121 |
|  | Melbourne, VIC, Australia, 3051 |
|  | St Albans, VIC, Australia, 3021 |
|  | Coffs Harbour, NSW, Australia, 2450 |
|  | Sydney, NSW, Australia, 2010 |
|  | Fortitude Valley, QLD, Australia, 4006 |
| Canada | Guelph, ON, Canada, N1G 0B4 |
|  | Québec, QC, Canada, G1N 4V3 |
|  | Québec, QC, Canada, G1V 4G2 |
|  | Sherbrooke, QC, Canada, J1J 2G2 |
|  | St-Charles-Borromée, QC, Canada, J6E 2B4 |
|  | Truro, NS, Canada, B2N 1L2 |
|  | London-Ontario, ON, Canada, N5W 6A2 |
|  | Greater Sudbury, ON, Canada, P3C 1X3 |
|  | Québec, QC, Canada, G1V 4W2 |
|  | Toronto, ON, Canada, M4G 3E8 |
|  | New Westminster, BC, Canada, V3L 3W4 |
|  | Victoria, BC, Canada, V8V 4A1 |
| Germany | Berlin, Germany, 10787 |
|  | Berlin, Germany, 10117 |
|  | Berlin, Germany, 13347 |
|  | Essen, Germany, 45355 |
|  | Mainz, Germany, 55116 |
|  | Wallerfing, Germany, 94574 |
|  | Weinheim, Baden-Wuerttemberg, Germany, 69469 |
|  | Witten, Germany, 58455 |
|  | Würzburg, Germany, 97070 |
| Japan | Ibaraki, Japan, 300-0062 |
|  | Tokyo, Japan, 180-0022 |
|  | Kanagawa, Japan, 211-0041 |
|  | Tokyo, Japan, 155-0031 |
| South Africa | Cape Town, South Africa, 7530 |
|  | Reiger Park, South Africa, 1459 |
|  | Cape Town, South Africa, 7700 |
|  | Johannesburg, South Africa, 2113 |
| United States | Charlottesville, VA, United States, 22911 |
|  | Hialeah, FL, United States, 33012 |
|  | Knoxville, TN, United States, 37909 |
|  | Lexington, KY, United States, 40509 |
|  | North Miami, FL, United States, 33173 |
|  | Orlando, FL, United States, 32806 |
|  | Rochester, NY, United States, 14609 |
|  | Silver Spring, MD, United States, 20904 |
|  | Wenatchee, WA, United States, 98801 |
|  | Walnut Creek, CA, United States, 94598 |
|  | Phoenix, AZ, United States, 85284 |
|  | DeSoto, TX, United States, 75115 |
|  | North Hollywood, CA, United States, 91606-3287 |
|  | Oakland, CA, United States, 94610 |
|  | Oklahoma City, OK, United States, 73111 |
|  | Glendale, AZ, United States, 85308 |

**Supplementary Table 2. Demographic and baseline characteristics of study participants, per-protocol set at Visit 2**

| **Characteristic** | **At-risk 18–49 group**  **N=393** | **≥60 group**  **N=417** |
| --- | --- | --- |
| Mean (SD) age at vaccination, y | 38.7 (7.8) | 68.5 (5.7) |
| Age category, n (%) |  |  |
| 18–49 y | 393 (100) | - |
| 60–69 y | - | 242 (58.0) |
| ≥70 y | - | 175 (42.0) |
| ≥80 y | - | 17 (4.1) |
| Female sex, n (%) | 230 (58.5) | 215 (51.6) |
| Race, n (%) |  |  |
| Asian | 46 (11.7) | 46 (11.0) |
| Black/African American | 72 (18.3) | 54 (12.9) |
| White | 244 (62.1) | 296 (71.0) |
| Other races^a^ | 31 (7.9) | 21 (5.0) |
| Ethnicity |  |  |
| Not Hispanic or Latino | 345 (87.8) | 388 (93.0) |
| Other ethnicities^b^ | 48 (12.2) | 29 (7.0) |
| Mean (SD) BMI, kg/m^2^ | 32.0 (8.7) | 29.0 (6.2) |
| Smoking status for tobacco |  |  |
| Current smoker | 75 (19.1) | 53 (12.7) |
| Former smoker | 75 (19.1) | 162 (38.8) |
| Never smoker | 243 (61.8) | 202 (48.4) |
| Smoking status for e-cigarettes |  |  |
| Current smoker | 23 (5.9) | 5 (1.2) |
| Former smoker | 17 (4.3) | 5 (1.2) |
| Never smoker | 352 (89.6) | 406 (97.4) |
| Unknown | 1 (0.3) | 1 (0.2) |
| Chronic disease of interest^c^ |  |  |
| 1 chronic disease of interest | 239 (60.8) | 148 (35.5) |
| ≥2 chronic diseases of interest | 154 (39.2) | 125 (30.0) |
| Cardiopulmonary conditions | 215 (54.7) | 132 (31.7) |
| Diabetes mellitus | 198 (50.4) | 126 (30.2) |
| Other chronic diseases of interest | 138 (35.1) | 126 (30.2) |

At-risk 18–49 group, group of participants aged 18–49 years at increased risk of respiratory syncytial virus (RSV) disease; ≥60 group, group of participants aged ≥60 years; N, number of participants in the per-protocol set at Visit 2; SD, standard deviation; y, years; n (%), number (percentage) of participants in a given category; BMI, body mass index.

^a^ Includes American Indian/Alaska Native, Native Hawaiian/other Pacific Islander, multiple race categories, race not reported, or race unknown.

^b^ Includes Hispanic or Latino, ethnicity not reported, or ethnicity unknown.

^c^ Chronic conditions of interest refer to the pre-defined conditions that are known to increase the risk for RSV disease: chronic obstructive pulmonary disease (Global Initiative for Chronic Obstructive Lung Disease grade 2–4), asthma (on maintenance and reliever therapy or frequent rescue treatment), cystic fibrosis, lung fibrosis, restrictive lung disease, interstitial lung disease, emphysema, bronchiectasis, chronic heart failure (New York Heart Association class II or higher), coronary artery disease, cardiac arrhythmia (requiring medical support), diabetes mellitus types 1 and 2 (with active treatment for at least the past 6 months), chronic kidney disease (G2-G3), chronic liver disease (moderate to severe), and active or chronic neurological/neuromuscular conditions.

**Supplementary Table 3. Demographic and baseline characteristics of study participants, per-protocol set for cell-mediated immunogenicity subset at Visit 2**

| **Characteristic** | **At-risk 18–49 group**  **N=72** | **≥60 group**  **N=44** |
| --- | --- | --- |
| Mean (SD) age at vaccination, y | 39.0 (8.1) | 68.0 (4.8) |
| Age category, n (%) |  |  |
| 18–49 y | 72 (100) | - |
| 60–69 y | - | 23 (52.3) |
| ≥70 y | - | 21 (47.7) |
| ≥80 y | - | 0 |
| Female sex, n (%) | 43 (59.7) | 26 (59.1) |
| Race, n (%) |  |  |
| White | 54 (75.0) | 38 (86.4) |
| Other races^a^ | 18 (25.0) | 6 (13.6) |
| Ethnicity |  |  |
| Not Hispanic or Latino | 57 (79.2) | 36 (81.8) |
| Other ethnicities^b^ | 15 (20.8) | 8 (18.2) |
| Mean (SD) BMI, kg/m^2^ | 30.4 (7.6) | 29.0 (5.1) |
| Smoking status for tobacco |  |  |
| Current smoker | 12 (16.7) | 3 (6.8) |
| Former smoker | 16 (22.2) | 20 (45.5) |
| Never smoker | 44 (61.1) | 21 (47.7) |
| Smoking status for e-cigarettes |  |  |
| Current smoker | 3 (4.2) | 0 |
| Never smoker | 69 (95.8) | 44 (100) |
| Chronic disease of interest^c^ |  |  |
| 1 chronic disease of interest | 37 (51.4) | 17 (38.6) |
| ≥2 chronic diseases of interest | 35 (48.6) | 9 (20.5) |
| Cardiopulmonary conditions | 49 (68.1) | 12 (27.3) |
| Diabetes mellitus | 28 (38.9) | 6 (13.6) |
| Other chronic diseases of interest | 32 (44.4) | 13 (29.5) |

At-risk 18–49 group, group of participants aged 18–49 years at increased risk of respiratory syncytial virus (RSV) disease; ≥60 group, group of participants aged ≥60 years; N, number of participants in the per-protocol set for cell-mediated immunogenicity subset at Visit 2; SD, standard deviation; y, years; n (%), number (percentage) of participants in a given category; BMI, body mass index.

^a^ Includes American Indian/Alaska Native, Asian, Black/African American, Native Hawaiian/other Pacific Islander, multiple race categories, race not reported, or race unknown.

^b^ Includes Hispanic or Latino, ethnicity not reported, or ethnicity unknown.

^c^ Chronic conditions of interest refer to the pre-defined conditions that are known to increase the risk for RSV disease: chronic obstructive pulmonary disease (Global Initiative for Chronic Obstructive Lung Disease grade 2–4), asthma (on maintenance and reliever therapy or frequent rescue treatment), cystic fibrosis, lung fibrosis, restrictive lung disease, interstitial lung disease, emphysema, bronchiectasis, chronic heart failure (New York Heart Association class II or higher), coronary artery disease, cardiac arrhythmia (requiring medical support), diabetes mellitus types 1 and 2 (with active treatment for at least the past 6 months), chronic kidney disease (G2-G3), chronic liver disease (moderate to severe), and active or chronic neurological/neuromuscular conditions.

**Supplementary Table 4. Adverse events of special interest reported from vaccination until study end, all vaccinated participants**

| **Event** | **Age onset (y)** | **Gender** | **Day onset** | **Resolved** | **Duration (days)** | **Study group** | **Vaccine-related^a^** | **SAE** | **Intensity grading^a^** | **Additional notes** |
| --- | --- | --- | --- | --- | --- | --- | --- | --- | --- | --- |
| ***Potential immune-mediated disease^a^*** | | | | | | | | | | |
| Hematoma (exacerbated) | 31 | Male | 14 | Yes | 217 | At-risk 18–49 group | Yes | No | Mild | Worsening of pre-existing condition. The participant had a medical history of type I diabetes mellitus. An hematoma initially developed at the insulin injection site 14 days post-vaccination and then aggravated all over the body (1–6 cm diameter), with the most prominent lesions in the abdominal region. Laboratory findings were normal (thrombocytes, hemoglobin, creatinine). A mildly prolonged activated partial thromobplastin time was noted, but no coagulopathy or bleeding disorder was identified. The investigator assessed the exacerbation of the hematoma as a potential immune-mediated disease that was non-serious (grade 1 intensity). The initial hematoma at the insulin injection site was not considered vaccine-related by the investigator, but the exacerbation was deemed vaccine-related. Overall, clinical and laboratory findings did not suggest an auto-immune etiology. |
| Type 1 diabetes mellitus (exacerbated) | 26 | Female | 37 | Yes | 1 | At-risk 18–49 group | No | Yes | Severe | Worsening of pre-existing condition. The participant had a medical history of type I diabetes mellitus, hypertension, gastritis, and hyperlipidemia. The investigator assessed the event as serious (grade 3 intensity) and considered that there was no reasonable possibility that it was vaccine-related. Another possible cause was infection. |
| Pernicious anemia | 74 | Female | 135 | No | - | ≥60 group | No | No | Mild | The event was discovered through a blood draw; the patient did not report symptoms. The investigator considered that there was no reasonable possibility that the event was vaccine-related. No etiology or underlying cause was known. |
| ***Atrial fibrillation^a^*** | | | | | | | | | | |
| Paroxysmal atrial fibrillation (exacerbated) | 45 | Male | 6 | Yes | 4 | At-risk 18–49 group | No | No | Mild | Worsening of pre-existing condition. The event was not confirmed by electrocardiography. The participant was obese (BMI ≥30kg/m^2^), had a relevant medical history of paroxysmal atrial fibrillation, paroxysmal supraventricular tachycardia, hypercholesterolemia, insulin resistance, and obstructive sleep apnea. The event did not result in changes to medication regimen taken for atrial fibrillation prior to study vaccination, was self-limited, and resolved spontaneously after 4 days of its onset. The investigator assessed the event as non-serious (grade 1 intensity) and considered that there was no reasonable possibility that it was vaccine-related. |
| Atrial fibrillation | 75 | Female | 131 | No | - | ≥60 group | No | Yes | Moderate | The event was confirmed by electrocardiography. The participant was obese (BMI ≥30kg/m^2^), had a relevant medical history of benign arrhythmia, obstructive sleep apnea, and family history of atrial fibrillation. The event was reported to be stable by the investigator. The investigator assessed the event as serious (grade 2 intensity) and considered that there was no reasonable possibility that it was vaccine-related. |

y, years; SAE, serious adverse event; at-risk 18–49 group, group of participants aged 18–49 years at increased risk of respiratory syncytial virus (RSV) disease; ≥60 group, group of participants aged ≥60 years; BMI, body mass index.

^a^ By investigator assessment.
